# A Challenge to the Ancient Origin of SIVagm Based on African Green Monkey Mitochondrial Genomes

**DOI:** 10.1371/journal.ppat.0030095

**Published:** 2007-07-06

**Authors:** Joel O Wertheim, Michael Worobey

**Affiliations:** Department of Ecology and Evolutionary Biology, University of Arizona, Tucson, Arizona, United States of America; The Pennsylvania State University, United States of America

## Abstract

While the circumstances surrounding the origin and spread of HIV are becoming clearer, the particulars of the origin of simian immunodeficiency virus (SIV) are still unknown. Specifically, the age of SIV, whether it is an ancient or recent infection, has not been resolved. Although many instances of cross-species transmission of SIV have been documented, the similarity between the African green monkey (AGM) and SIVagm phylogenies has long been held as suggestive of ancient codivergence between SIVs and their primate hosts. Here, we present well-resolved phylogenies based on full-length AGM mitochondrial genomes and seven previously published SIVagm genomes; these allowed us to perform the first rigorous phylogenetic test to our knowledge of the hypothesis that SIVagm codiverged with the AGMs. Using the Shimodaira–Hasegawa test, we show that the AGM mitochondrial genomes and SIVagm did not evolve along the same topology. Furthermore, we demonstrate that the SIVagm topology can be explained by a pattern of west-to-east transmission of the virus across existing AGM geographic ranges. Using a relaxed molecular clock, we also provide a date for the most recent common ancestor of the AGMs at approximately 3 million years ago. This study substantially weakens the theory of ancient SIV infection followed by codivergence with its primate hosts.

## Author Summary

Elucidating the factors that influence the emergence of viral pathogens is of great importance to the study of infectious disease. HIV is understood to have originated from simian immunodeficiency viruses (SIVs) infecting nonhuman African primates, but the length of time the virus has been present in these apes and monkeys is not known. These infected primates do not normally develop immunodeficiency, and understanding the age of SIV might help explain why. It has been suggested that some of these monkeys have been infected for millions of years, because many closely related monkey species are infected with closely related viruses. One of the most prominent examples of this relationship is between the African green monkeys and their SIVs. In this study, we compared viral phylogenetic trees to those of their hosts' mitochondrial genomes and found that they do not support the theory of ancient infection followed by codivergence. Our results suggest that SIV did not infect these monkeys until after speciation and subsequently swept across their geographical ranges. If this infection is relatively recent, then avirulence may have evolved over a shorter time frame than previously suggested. This finding could have implications for the future trajectory of HIV disease severity.

## Introduction

More than 30 nonhuman primate species in sub-Saharan Africa are naturally infected with simian immunodeficiency virus (SIV) [1]; however, the evolutionary forces shaping SIV diversity remain unclear. One of the most important unanswered questions regarding SIV evolution is whether it is an ancient infection that has been codiverging with its primate hosts for millions of years, or whether the virus may have arrived more recently and swept across already established primate lineages. Codivergence of viruses with their hosts has been inferred in other cases [2,3], including other retroviruses [4,5], where a close match between the host and viral phylogenetic trees suggests an ancient association. Furthermore, recent genomic analysis suggests that endogenous lentiviruses may have been infecting mammals for the last 7 million years [6]. Although it now seems clear that the overall pattern of the SIV and host phylogenies cannot be reconciled with a simple history of codivergence [7], certain groups of SIVs and their hosts seem to suggest a shared evolutionary history.

Among the SIV taxa, perhaps the best candidate for codivergence is the African green monkey (AGM) clade and their viruses, SIVagm. The AGM genus, *Chlorocebus,* consists of four species *(C. aethiops, C. pygerythrus, C. sabaeus,* and *C. tantalus),* each with its own corresponding SIV lineage (SIVgri, SIVver, SIVsab, and SIVtan) [8–11]. The monkeys are geographically distributed across sub-Saharan Africa, with *C*. *sabaeus* in West Africa, *C*. *tantalus* in central Africa, C. aethiops (grivet) in northeastern Africa, and *C*. *pygerythrus* (vervet) ranging from East to southern Africa [12]. Studies using mitochondrial 12s rRNA have demonstrated monophyly among most AGM species (i.e., each individual shares a common ancestor more recently with every member of its own species than with any other AGM species) [13,14]. However, 12s analysis provides very low statistical support for the branching order among the AGM taxa, and these studies were unable to resolve whether *C*. *pygerythrus* from Tanzania and South Africa are monophyletic or paraphyletic.

On the face of it, the fact that this monophyletic clade of primates is infected by SIVs that also form a monophyletic clade provides compelling evidence of codivergence; however, a degree of caution is warranted whenever such inferences are made. An alternative mechanism by which pathogen and host topologies could resemble each other is preferential host-switching [7]. This model proposes that viruses are more likely to be transmitted between hosts with less phylogenetic distance separating them. This will lead to a viral phylogeny that is similar to the host tree, even in the absence of shared history.

There is ample evidence demonstrating that SIV can switch hosts, with many examples of natural cross-species transmission of SIV among primates. SIVagm has been transmitted to the closely related patas monkey [15] and the more distantly related yellow and chacma baboons [16,17]. Furthermore, two distinct viral lineages infecting chimpanzees (and possibly gorillas) [18,19] and sooty mangabeys [20] have been introduced into the human population at least 11 times, giving rise to HIV [21]. In captivity, SIVagm has been transmitted to the African white-crowned mangabey [22], and SIV from sooty mangabeys has been transmitted to several macaque species [23,24]. The relationships among SIVs are further complicated because many viruses, such as those infecting chimpanzees, sabaeus monkeys, mandrills, and Dent's Mona monkeys, represent recombinant lineages whose origins must have involved cross-species transmissions of SIV [25–28].

Nevertheless, additional evidence in favor of AGM–SIVagm codivergence has been put forward. The codivergence hypothesis predicts not only that the AGM species will share closely related SIVs, but also that the branching order within the virus clade and monkey clade should match. Such congruence has been reported from an analysis of the AGM *CD4* gene [29], which suggested phylogenetic congruence between this nuclear marker and the SIVagm *env* gene. However, the trees inferred for both virus and host genes were not well supported. Another study involving a nuclear gene, *CCR5,* which codes for a coreceptor SIV uses to gain entry into host cells, concluded that coevolution between SIV and AGMs had occurred, implying an ancient infection [30].

More generally, the fact that primates naturally infected with SIV do not normally develop immunodeficiency seems to indicate a lengthy host–virus association. Prevalence of the virus in adult AGMs has been documented in excess of 70% [31,32]. Despite continuous viral replication, which can reach titers comparable to those found in humans infected with HIV [33,34], immunodeficiency has only been observed once in an AGM that was co-infected with another retrovirus, STLV-I [35]. On the other hand, SIVagm is lethal when transmitted to non-African host monkeys such as the pigtailed macaque [36,37]. The low virulence observed in the natural host (AGMs), however, does not necessarily indicate millions of years of evolution in response to SIV infection. Fossil evidence and genetic diversity studies propose that the AGM clade is on the order of millions of years old [38,39], whereas molecular clock calculations have inferred a date of the most recent common ancestor (MRCA) of SIVagm at only hundreds or thousands of years old [40]. Estimates of such a recent origin of SIVagm cannot be dismissed simply on the basis of the observation that SIVagm is relatively benign in its natural hosts.

The purpose of this study was to perform a rigorous phylogenetic test of the hypothesis of ancient codivergence between the AGMs and their SIVs. To do so, we sequenced complete AGM mitochondrial genomes, an approach that has produced what is, to our knowledge, the first statistically well-resolved AGM phylogeny. In comparing this phylogeny to ones inferred from SIVagm genomes, we found that the viral genome topology and host mitochondrial topology were incongruent and therefore provided no support for an ancient infection followed by codivergence.

## Results

### SIVagm Phylogenies

**Figure 1:**
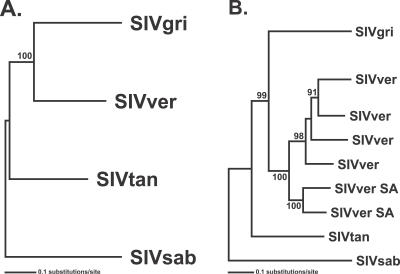
Phylogenetic Relationships among SIVagm Genomes (A) SIVagm genomes and (B) SIVagm genomes plus two SIVver *env* genes from South Africa (SA). Both trees are shown with SIVsab as an outgroup, although midpoint rooting produces the same rooting pattern. ML nonparametric bootstrap support values (>50%) are shown on nodes.

We constructed the maximum likelihood (ML) SIVagm phylogeny using four previously published SIVagm genomes, one from each named species. The inferred phylogeny placed SIVgri (grivet) and SIVver (vervet) together with high bootstrap support (Figure 1A). Midpoint rooting indicated that SIVsab was the most basal taxon. To test the robustness of our ML topology, we performed the Shimodaira–Hasegawa test (SH-test) [41] on all three possible unrooted SIVagm topologies. Using this conservative test, we were able to reject both alternative SIVagm unrooted topologies (*p* < 0.05) (Table 1).

**Table 1:**
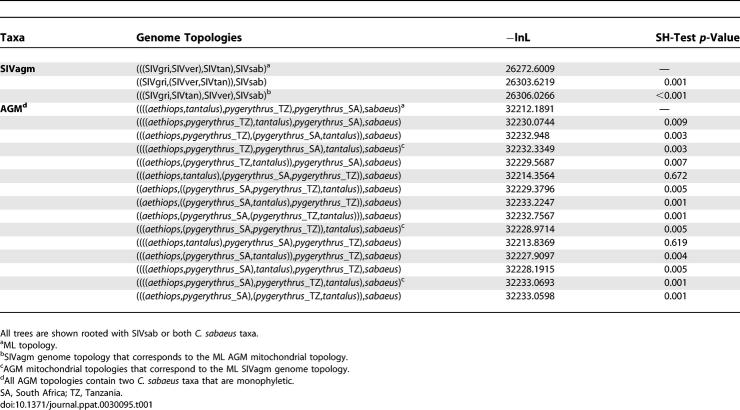
Shimodaira-Hasegawa Test on SIVagm and AGM Mitochondrial Phylogenies

To ensure that this pattern of SIVgri and SIVver forming a monophyletic clade was consistent for a larger sample of SIVagm strains, we also constructed a phylogeny using all seven available SIVagm genomes plus the complete *env* gene from two SIVver taxa that were isolated from *C*. *pygerythrus* in South Africa. We decided to include these subgenomic sequences because the complete SIVver genomes were all isolated from *C*. *pygerythrus* from East Africa, and we desired a better geographic representation of SIVver samples. Using this dataset, we recovered the same species-specific topology, with SIVgri and SIVver clustering together with strong support and SIVtan falling basal when rooted with SIVsab (Figure 1B). All SIVver taxa form a monophyletic clade.

### AGM Nuclear Loci

**Figure 2:**
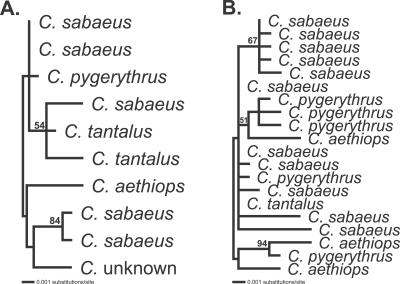
Phylogenetic Relationships among AGM Nuclear Loci (A) *CD4* phylogeny and (B) *CCR5* phylogeny. Both trees are midpoint rooted. ML nonparametric bootstrap support values (>50) are shown on nodes. “*C*. unknown” in (A) refers to a taxon with no published species-specific information.

To determine if available sequence data were sufficient to infer the branching order among the AGM species, we constructed phylogenies using the *CD4* and *CCR5* genes. Although available 12s rRNA data have proven useful for differentiating AGM species, they were not sufficient for resolving the phylogeny with statistical confidence. Furthermore, additional nuclear gene data have accumulated recently but have not yet been subjected to phylogenetic analysis. Despite earlier studies with fewer sequences, which seemed to determine the AGM topology, our results with the most complete alignments of nuclear gene sequences indicated that coding nuclear loci do not sufficiently resolve the AGM phylogeny. According to the *CD4* topology, AGM species are not reciprocally monophyletic (Figure 2A). There is low bootstrap support across the entire *CD4* tree. We were also unable to resolve the branching order using *CCR5* (Figure 2B). All AGM species for which more than one *CCR5* allele was analyzed exhibited paraphyly. Moreover, the only *CCR5* allele from C. tantalus is identical to one of the C. sabaeus alleles, implying that *CCR5* is not useful in distinguishing AGM species, let alone their phylogenetic relationships.

### Mitochondrial Phylogenies

**Figure 3:**
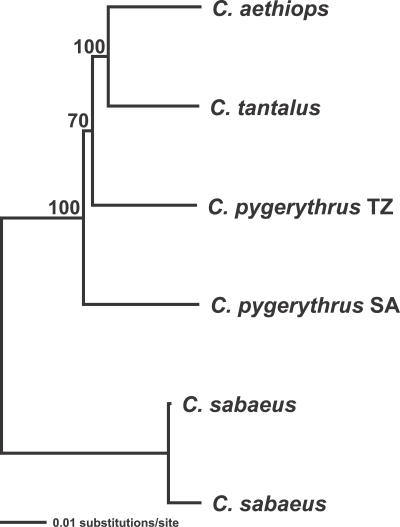
Phylogenetic Relationships among AGM Mitochondrial Genomes ML tree is shown with C. sabaeus taxa as an outgroup, although midpoint rooting produced the same rooting pattern. C. pygerythrus were isolated from Tanzania (TZ) and South Africa (SA). ML nonparametric bootstrap support values (>50%) are shown on nodes.

**Figure 4:**
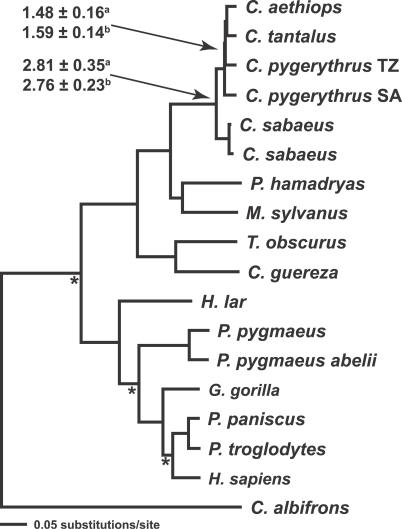
Relaxed Molecular Clock Analysis of Catarrhine Taxa with Estimated AGM Divergence Dates ML tree is rooted using C. albifrons as an outgroup, with mean MRCA date for the AGM taxa and 95% confidence intervals estimated from 100 replicate trees from (a) ML bootstrap analysis and (b) Bayesian MCMC analysis. Asterisks designate nodes as being fossil-constrained as defined by Raaum et al. [42].

To generate a sequence alignment likely to have sufficient phylogenetic signal to resolve the AGM phylogeny with a high degree of confidence, we sequenced complete mitochondrial genomes—an approach that has yielded robust phylogenies for other primates [42]—for *C. sabaeus, C*. *tantalus,* and *C*. *pygerythrus* from Tanzania and South Africa. Using an ML framework, we constructed a phylogeny comprised of these four genomes plus the previously published *C*. *aethiops* and C. sabaeus mitochondrial genomes. We inferred a single best topology that placed *C*. *aethiops* and *C*. *tantalus* together with high bootstrap support (Figure 3); however, we were unable to resolve the phylogenetic relationship between the two *C*. *pygerythrus* taxa. While the ML tree indicated these two taxa are paraphyletic, with the taxon from South Africa branching off before the one from Tanzania, there is low bootstrap support for this inference. Of interest, the corrected genetic distance (GTR + Γ_4_) between the two *C*. *pygerythrus* taxa was greater than that between *C*. *tantalus* and *C*. *aethiops*. Both midpoint and outgroup rooting using additional mitochondrial genomes (Figure 4) placed *C*. *sabaeus* as the most basal AGM taxon. A topology identical to the one inferred via ML in PAUP* was also inferred in a Bayesian framework. This tree placed C. aethiops and C. tantalus together with a posterior probability of 1.0.

We then compared the unrooted AGM topologies using the SH-test, which rejected all AGM mitochondrial topologies that do not place *C*. *tantalus* with *C*. *aethiops* and *C*. *sabaeus* with *C*. *pygerythrus* (Table 1). However, we were unable to reject either of the alternate arrangements within *C*. *pygerythrus,* in which the Tanzanian *C*. *pygerythrus* branched first before the taxon from South Africa, or where the two *C*. *pygerythrus* taxa formed a monophyletic clade.

### Dating the AGM Origin and Radiation

Using a penalized likelihood approach [43], we estimated the age of both the AGM MRCA and the subsequent radiation of *C. aethiops, C. tantalus,* and C. pygerythrus (Figure 4). This analysis was performed using trees generated under ML and Bayesian Markov-chain Monte Carlo (MCMC) frameworks. According to the ML analysis, the AGM lineages shared an MRCA 2.81 ± 0.35 million years ago (MYA); *C. aethiops, C*. *tantalus,* and C. pygerythrus shared an MRCA 1.48 ± 0.16 MYA. Estimates from the MCMC analysis did not differ considerably, placing the AGM common ancestor at 2.76 ± 0.23 MYA and the radiation at 1.59 ± 0.14 MYA. Because uniform branching order among the *C. aethiops, C. tantalus,* and two C. pygerythrus lineages was not observed in the ML analysis, we were unable to estimate the date of divergence events among these species. Our dates of other divergence events among the catarrhine species did not differ appreciably from those presented by Raaum et al. [42] and are therefore not reported here.

### Test of Phylogenetic Congruence

As an explicit test for host–viral phylogenetic congruence, which, if present, would be a strong indication of codivergence, we used a series of SH-tests to determine if the SIVagm and AGM mitochondrial phylogenies were significantly different from each other (Table 1). First, we compared the ML SIVagm topology to the SIVagm topology that corresponded to the ML AGM mitochondrial topology (labeled footnote b in Table 1). The SH-test on the SIVagm genomes rejected this alternate topology (*p* < 0.05). Hence, there is convincing evidence that the SIVagm genomes did not evolve along the same topology as the AGM mitochondrial genomes. Of note, the test also rejected the alternate SIVagm topologies when the first 3,500 bases, the recombinant region in SIVsab, were included (*p* < 0.05); thus, these results are not affected by the recombinant origin of SIVsab.

Finally, we compared the ML AGM mitochondrial topology to the three AGM mitochondrial topologies that corresponded to the ML SIVagm topology (labeled footnote c in Table 1). We made these three comparisons because of the ambiguity that exists in the branching order of the two *C*. *pygerythrus* taxa. All three of these topologies were rejected by the SH-test on the AGM mitochondrial dataset (*p* < 0.05). In other words, we can confidently reject the hypothesis that the AGM mitochondrial genomes evolved along the same topology as the SIVagm genomes.

## Discussion

Our results present a significant challenge to the ancient origin of SIVagm followed by codivergence with their AGM hosts. Using an ML framework, we inferred robust phylogenies from the AGM mitochondrial genomes and SIVagm genomes. Although *C*. *sabaeus* are the most basal taxa for both the mitochondrial genome and SIVagm trees (according to midpoint rooting methods), the other taxa do not share the same topology. As in previous studies on AGM taxonomy, we were unable to determine if *C*. *pygerythrus* from Tanzania and South Africa form a monophyletic clade, even though they are infected by the same SIVagm. Given the genetic distance between them, if *C*. *pygerythrus* is monophyletic, then it exhibits greater genetic diversity than is observed between C. tantalus and C. aethiops. In any case, using the SH-test we can confidently state that the virus did not evolve along the topology of the host mitochondrial genomes, and conversely, that the mitochondrial genomes did not evolve along the viral topology.

These results demonstrate the usefulness of complete mitochondrial genomes in resolving recent primate divergence events, even those that occurred within a short span of time as with *C*. *pygerythrus, C*. *tantalus,* and *C*. *aethiops*. We also present the first date for the diversification of AGMs that accounts for the rate variation across the catarrhine phylogeny. The dating of the AGM MRCA at 2.81 ± 0.35 MYA indicates that if SIVagm did codiverge with its hosts (which seems unlikely given our findings), it must have infected the AGM common ancestor nearly 3 MYA.

**Figure 5:**
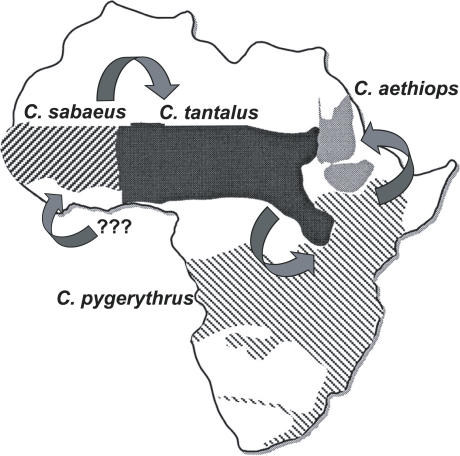
Hypothesized SIVagm Transmission Pattern across sub-Saharan Africa AGM distributions across the African continent are depicted. According to SIVagm phylogenetic analysis, C. sabaeus was the first AGM to be infected with SIV, although the source of this infection is unknown. The arrows depict a possible route of transmission of the virus across already established AGM ranges. It should be noted that the inferred SIVagm phylogeny does not distinguish between the depicted route of transmission and a route in which C. tantalus first infected *C. aethiops,* which in turn infected C. pygerythrus. This figure is modified from Beer et al. [64], which utilized the range map from Lernould [12].

Without evidence for a shared history, a model other than codivergence is needed to explain the observed pattern of SIVagm infection. A preferential host-switching model [7], whereby viral transmission occurred over already established primate host ranges and favored the cross-species transmission of SIV from an initial AGM population to others, is a strong candidate. When the SIVagm phylogeny is mapped onto the distribution of AGMs in Africa, a geographic pattern of west-to-east transmission emerges (Figure 5). SIVsab is likely the most basal of the SIVagm taxa [1], and its host, *C*. *sabaeus,* has the westernmost geographic distribution. SIVtan branches off next, and the range of *C*. *tantalus* begins at the Volta River, just east of the current *C*. *sabaeus* range, and continues into central Africa [12]. Finally, SIVgri and SIVver are the most derived SIVagm taxa and infect monkeys inhabiting the easternmost part of the continent. Since SIVagm is predominantly a sexually transmitted virus [32], and AGM species are known to hybridize in the wild [12], sexual encounters between AGM species may have facilitated SIV transmission at the edges of AGM ranges and the subsequent geographic spread of the virus.

While geography and behavior likely provided ample opportunity for the transmission of an initial SIVagm variant from one AGM species to the next, these factors alone cannot explain why the various AGM species would have acquired SIV *only* from other AGM species, rather than other infected primate species. We speculate that intrinsic immunity factors, such as the APOBEC proteins [44–46] and TRIM5α[47], may have played a role in this context. These proteins have been shown to prevent initiation of retroviral infection via a variety of mechanisms [48]. Specifically, these factors may have prevented the more distantly related *Cercopithecus* monkeys from becoming infected with SIVagm and similarly blocked the introduction of SIV from non-AGM species into the AGMs. In this light, the infection of the patas monkey with SIVsab might be an important clue, since this monkey is very closely related to the AGMs [49] and has been observed engaging in aggressive behavior with C. sabaeus [15]. Although the findings presented here on the age of SIV suggest that the virus was not a relevant force in the ancient evolution of these proteins, intrinsic immunity factors may have been crucial in shaping the distribution of SIV across the range of the African primates it infects.

It is important to bear in mind that mitochondrial genomes, despite their length and phylogenetic information, represent only a single maternally inherited genetic locus. Regions of the AGM nuclear genome may have evolved along different evolutionary trajectories, some of which might be congruent with the viral evolutionary history. Furthermore, the AGM mitochondria may have experienced incomplete lineage sorting during the speciation events, which could obscure the species tree [50,51]. Nevertheless, our results represent the first and only statistically robust species-level AGM phylogeny to our knowledge, and this phylogeny unequivocally disagrees with the viral phylogeny. While there are examples of incongruence among mitochondrial and nuclear markers in the African guenons [52], it is unlikely that other population level phenomenon such as introgression would occur in the mitochondria of the AGMs. The strong philopatry observed in AGM females, coupled with a dominance hierarchy that discourages breeding with migrant females, would decrease the likelihood of reproductive success of a female immigrant and therefore the probability of mitochondrial introgression [49]. In future studies, the inclusion of additional AGM mitochondrial genomes and other informative nuclear loci would be useful in determining if any of these population level phenomena have obscured the evolutionary history of the AGM. Nevertheless, we are confident that this study poses a significant challenge to the theory of ancient infection and codivergence.

Given the conflicting AGM mitochondrial and SIVagm topologies presented here, the case for codivergence between AGMs and their SIVs is limited to the observations that (1) C. sabaeus and SIVsab are the basal taxa in the mitochondrial and SIV phylogenies, respectively, and (2) SIVagm forms a monophyletic group. However, the fact that C. sabaeus is basal in the mitochondrial phylogeny can hardly be used to argue in favor of codivergence when the remainder of the host phylogeny differs significantly from the viral one. In light of our findings, the ancient codivergence model is, to us, a less parsimonious explanation of the observed patterns than a preferential host-switching model with a relatively recent origin of SIVagm. In the absence of evidence in favor of AGM–SIVagm codivergence, we are left to wonder about the case for codivergence in other African monkeys infected with SIV.

A recent ancestry of SIVagm calls into question the conclusions put forth by Kuhmann et al. [30] regarding the coevolution of SIVagm with host protein CCR5. Our analysis of the *CCR5* locus suggests that there are no unique species-specific differences among the alleles that would suggest coevolution. Furthermore, the study by Kuhmann et al*.* did not perform a formal test for selection and assumed that a higher proportion of nonsynonymous-to-synonymous substitutions was evidence of positive selection; however, the ratio they observed (approximately two nonsynonymous changes for every one synonymous change) is consistent with purifying selection, as nonsynonymous mutations are more frequent by chance alone.

If SIVagm is not the result of an ancient infection, then its avirulence in its natural hosts may have evolved over a much shorter time frame than implied by the ancient codivergence model. Competition experiments by Ariën et al. [53] between matched pairs of HIV samples from 2002–2003 and the late 1980s suggest that the virus may be attenuating in the human population. The authors proposed that this loss of replicative fitness by HIV might be due to its adaptation to the human immune system coupled with repeated bottlenecks resulting from human-to-human transmission. Their data suggest that evidence of reduced virulence could be perceived in relatively short periods of time. Precise dating of the original SIV infection in the AGMs may help us better appreciate the evolutionary time frame in which such change is possible in the viral lineage.

## Materials and Methods

### Mitochondrial genome amplification and sequencing.

DNA extracts from *C*. *pygerythrus* from Tanzania (CAE9649), *C*. *pygerythrus* from South Africa (V389), *C*. *sabaeus* (Letta), and *C*. *tantalus* (Bébé) were provided by A. C. van der Kuyl. Mitochondrial genomes were amplified using three PCR primer sets whose products ranged from 5 to 8 kilobases, which were designed based on the method developed by Raaum et al*.* [42]. Reactions were performed using the TripleMaster PCR system with an annealing temperature of 52 °C with an extension time of 9 min for the first ten cycles, which was extended 15 s for each additional cycle. Each reaction was run for 35 cycles. PCR products were purified using QIAquick PCR purification kits (Qiagen, http://www.qiagen.com/), and the templates were sequenced using internal primers. Regions that proved difficult to sequence from original template were re-amplified using internal PCR primers and then sequenced using those primers. All primer sequences are shown in Table S1. PCR reactions were confirmed using a 0.8% agarose gel stained with SYBR Safe (Invitrogen, http://www.invitrogen.com/). DNA sequencing was performed by the Genomic Analysis and Technology Core Facility (University of Arizona, Tucson, Arizona, United States) using an automated sequencer (Applied Biosystems 3730XL DNA Analyzer, http://www.appliedbiosystems.com/) until each base had been sequenced at least twice. Contigs were then assembled using Sequencher version 4.2 (Gene Codes Corporation, http://www.genecodes.com/).

Each of the four mitochondrial genomes was completely sequenced, except for *C*. *sabaeus,* for which a 200–base-pair region proved problematic, possibly due to secondary structure of the template. In addition, the mitochondrial genome of *C*. *tantalus* exhibited a repeat structure within its control region that, while unusual, is not unprecedented [54]. A 115–base-pair region was repeated as many as three instances in some sequencing reactions, whereas in others it appeared only a single time. PCR amplifications of the *C*. *tantalus* control region indicated that multiple forms of this region existed. Unfortunately, due to degradation of our original template, we were unable to determine whether this repeat structure was due to PCR error or actual heterogeneity in the sample. Nevertheless, this region was excluded from our analysis, because the repeats had no homologous region in any other mitochondrial genome and were therefore phylogenetically uninformative.

### Phylogenetic analyses.

*CD4* and *CCR5* sequences were downloaded from GenBank. *CD4* genes labeled as Barbados were classified as C. sabaeus based on recent genetic testing using cytochrome *b* sequence analysis [55]. All redundant *CCR5* sequences were removed, except for those that were isolated from different species. Each of these datasets was aligned by hand using Se-Al [56]. ML phylogenetic trees for these two loci were inferred using a heuristic search in PAUP* version 4.0b10 [57]. The models of nucleotide substitution, Kimura81 + Inv for *CD4* and HKY + Inv for *CCR5,* were identified by ModelTest version 3.7 [58]. Bootstrap support was assessed using 1,000 and 100 replicates for the *CD4* and *CCR5* topologies, respectively, using the ML nucleotide substitution parameters estimated from the ML phylogeny.

The four AGM mitochondrial genomes sequenced here and the previously published *C*. *aethiops* and C. sabaeus mitochondrial genomes were aligned by hand using Se-Al, except for the variable D-loop regions, which were aligned using CLUSTAL X [59]. A single phylogenetic tree was inferred using an exhaustive search with ML parameters inferred under a GTR + Γ_4_ nucleotide substitution model in PAUP*. Bootstrap support was assessed in an ML framework whereby the nucleotide substitution parameters were reestimated for each replicate and a heuristic search was performed; this was done for 1,000 replicates.

In addition, a phylogenetic tree was inferred with a GTR + Γ_4_ nucleotide substitution model in a Bayesian framework using MrBayes version 3.0 [60]. Two independent runs were performed, each using 1 million steps with four chains sampling every 100 steps. The first 10% of the trees were removed and posterior probabilities were calculated from these post-burnin trees.

SIVagm genomes were obtained from the HIV Sequence Database at Los Alamos National Laboratory (LANL, http://hiv.lanl.gov/content/hiv-db/mainpage.html). In the initial analysis, the four genomes were aligned using CLUSTAL X. We excluded the first 3,500 bases of all SIVagm genomes from our analyses, because SIVsab is a known recombinant in the 3′ part of this region, and its phylogenetic placement is ambiguous in the 5′ section of this region [25]. The sequences were aligned using CLUSTAL X, and an exhaustive search inferred a single phylogenetic tree using ML parameters estimated under a GTR + Γ_4_ model in PAUP*. In the secondary analysis on all seven published SIVagm genomes and the *env* genes of SIVver from South Africa, the sequences were also obtained from the LANL database. A single phylogenetic tree was found using a heuristic search with ML parameters inferred under a GTR + Γ_4_ model in PAUP*. Bootstrap support was assessed in an ML framework whereby each nucleotide substitution parameter was reestimated for each replicate and a heuristic search was performed; this was done for 1,000 replicates for the four-taxa tree and for 100 replicates for the nine-taxa tree.

The SH-test was performed in PAUP* on the unrooted bifurcating topologies for the six AGM mitochondrial genomes, in which the C. sabaeus taxa are monophyletic, and the four initial SIVagm genomes. The test parameters were estimated using a GTR + Γ_4_ model with 1,000 RELL replicates.

### Molecular clock.

Molecular clock analysis was carried out using the r8s software developed by Sanderson [61]. In order to estimate the divergence dates of and within the AGMs, we included other complete mitochondrial genomes from the Old World monkeys *Colobus guereza, Macaca sylvanus, Papio hamadryas,* and *Trachypithecus obscurus;* lesser and great apes *Hylobates lar, Gorilla gorilla, Homo sapiens, Pan paniscus, Pan troglodytes, Pongo pygmaeus pygmaeus,* and *Pongo pygmaeus abelii;* and a New World monkey, *Cebus albifrons,* which was used as an outgroup to root the phylogeny. An alignment of these mitochondrial genomes was obtained using CLUSTAL X. The two variable D-loop regions were removed from further analysis due to their poor sequence conservation.

Our analysis closely followed that of Raaum et al. [42], who first estimated divergence dates using many of the same primate mitochondrial genomes. We used a semiparametric approach with a penalized likelihood method in which the rate of evolution along each branch is allowed to vary, but a roughness penalty prevents the rate from varying too much from branch to branch [61]. An optimal smoothing parameter was chosen by cross-validation analysis. The non-clocklike behavior of this dataset was not unexpected given the decrease in the rate of evolution observed in apes [62,63]. We based our divergence estimates on three fossil-derived calibration points identified by Raaum et al.: the 6-MYA split between *Pan* and *Homo,* the 14-MYA split between the Asian great apes *(Pongo)* and the African great apes, and the 23-MYA split between hominoids and the Old World monkeys. These fossil-derived dates were entered into r8s as point estimates, rather than intervals, because r8s does not work well with narrow calibration windows.

To estimate confidence intervals for the age of the AGM clade and the radiation of *C. aethiops, C. tantalus,* and *C. pygerythrus,* we used ML branch lengths estimated from 100 nonparametric bootstrap replicate trees in PAUP* and 100 trees from a Bayesian MCMC run. Bootstrap trees in PAUP* were obtained using GTR + Γ_4_ parameters estimated from an ML tree. Trees from the MCMC run were sampled every 9,000 trees after the first 100,000 burnin trees. In both cases, every tree supported the identical topology for all taxa except *C. aethiops, C. tantalus,* and the two C. pygerythrus. The Bayesian analysis did, however, place C. aethiops and C. tantalus together 100% of the time, which is consistent with our previous phylogenetic analysis on the AGM mitochondrial genomes. We provide estimates of error as two standard deviations from the mean age of the estimated node for each of these datasets (ML and MCMC); these estimates are conservative, as they do not capture the uncertainty in the fossil record.

## Supporting Information

Table S1 PCR and Sequencing Primers (37 KB DOC)

### Accession Numbers

The GenBank (http://www.ncbi.nlm.nih.gov/Genbank/) accession numbers for the AGM mitochondrial genomes sequenced in this study are EF597500–EF59750. Accession numbers for other genes and genomes are as follows: previously published AGM mitochondrial genomes (AY863426 and DQ069713), *CCR5* (AB015944, AF035221, AF035222, AF035223, AF081577, AF105286, AF162006, AF162007, AF162016, AF162017, AF162020, AF162022, AF162023, AF162025, AF162026, AF162030, AF162031, AF252552, U83324, and U83325), *CD4* (AF001221–AF001228, D86589, and X73322), initial SIVagm genomes (M66437, L40990, U58991, and U04005), additional SIVagm genes and genomes (BD092095, M30931, M29975, AF015905, and AF015906), and additional primate mitochondrial genomes (AY863427, NC_002764, Y18001, AY863425, X99256, NC_001645, NC_001807, NC_001644, NC_001643, NC_001646, NC_002083, and AJ309866).

## Abbreviations

AGM: African green monkey
MCMC: Markov-chain Monte Carlo
ML: maximum likelihood
MRCA: most recent common ancestor
MYA: million years ago
SH-test: Shimodaira–Hasegawa test
SIV: simian immunodeficiency virus
