# Supplementary material for: A Challenge to the Ancient Origin of SIVagm Based on African Green Monkey Mitochondrial Genomes
Source: PLoS Pathog. 2007 Jul 6;3(7):e95. doi: 10.1371/journal.ppat.0030095 (PMC1904472; doi:10.1371/journal.ppat.0030095)
Supplement: Supplementary file 1 [file ppat.0030095.st001.doc]

Table S1. PCR and Sequencing Primers

| **Primer Name** | Primer Sequence | **Function** |
| --- | --- | --- |
| 125R | TGTCACTGCTGTTTCC | S |
| 204R | GCATGTGCGTGCTTGATACT | R,S |
| 218R | TCGGGTTAATCGTATGG | S |
| 318R | ACGAAATTGACCAACCCTTG | R,S |
| 574F | AGCAACAGCTTAAAACTCAAAGGA | S |
| 766R | ACGTTAGGTCAAGGTGTAGCTTATGAG | S |
| 788R | GCCCATCTTTTTCCATCTCA | S |
| 792R | GCCCATCTTTTTCCATCTCA | S |
| 927R | AGTATATTTGAGGGGAGTGACG | S |
| 978F | TATAGAGGAGATAAGTCGTAACA | S |
| 1076R | TGAGATCTTCCGGGTGTAGG | S |
| 1438F | CCTACCGAGCCTGGTGATAGCTGGT | S |
| 1660R | TGAGCTTGAACGCTTTCTTAATT | S |
| 1970F | CCCCGCCTGTTTACCAAAAACATCA | L,S |
| 2115R | ACGGACAGGTCAATTTCACTGG | S |
| 2361F | GCGCAATTCTATTCTAGAGTCC | S |
| 2442F | GCTATCAAGGGTTCGTTTGTTC | S |
| 2730R | CACGTAGGACTTTAATCGTTGAACAAACGAACC | L |
| 2909R | ATTGCATCGGCGAAWGGTTGTAGTA | S |
| 3039F | CCTGTTCATCCTAGCCATATCC | S |
| 3170F | GTCGCCCAAAYAATCTCATACGAA | S |
| 3321R | TAGTGT RGAG RTAAATCATATT | S |
| 3648R | CTGGAGATTGTAATGGGTGTTA | S |
| 3729F | GTCTGACAAAAGAGTTACTTTGATA | S |
| 3888R | TTTCGGGGTATGGGCCCGATAGCTT | S |
| 3902F | CCTAGCCCAACTCACCATCTAC | S |
| 3905R | GGGGGTTAATTAGTACGGGAAG | S |
| 3985F | AGCAGGCCTGGAAATAAACATA | S |
| 4228F | ACTTTTGAATTCCAGAGGTCAC | S |
| 4325R | GTTTTTGYCATGTGAG RAGGAGTAA | S |
| 4462F | CTCAATCACTCACATAGG | S |
| 5079R | ATTTGCGTTCAGTTGATGC RGAGTA | S |
| 5213F | AAAAGGCGGGAGAAGCCCCGGCAGA | S |
| 5226R | TGTAAACCTGAAGACAGAGGTC | S |
| 5275F | ACCYCTGTCTTTAGGTTTACAGCCTAATG | L,S |
| 5732F | ACAGGTTGAACAGTGTATCC | S |
| 6668F | CTATCCGGAATACCCCGACGCTACT | S |
| 6694R | GAGTAGCGTCGAGGTATTCCGGATA | L,S |
| 6936R | AGGATTGGCTTGAAACC | S |
| 7381F | GGACAYCAATGATACTGAACCTA | R,S |
| 7472R | ATACGDACAGGGGCTTCAATTGG | S |
| 7723F | CCACTAAAAATYTTYGAAATAGG | R,S |
| 7726R | GTTAACGCTACAATAGCTGTGC | S |
| 7945R | TAGTTGTCAGGGGTTATTGG | S |
| 8224F | TTACCACAACTAACC | S |
| 8502F | CCA RTAGCCYTAGCCGTACG | S |
| 8622R | AGTGTGAAAACATAGG | R,S |
| 8915F | CGAGAAAGCACCTACCAAGGCCA | S |
| 8958R | AGGCTTGAGTGGTAGAATGCTC | S |
| 9461R | ACATCTACAAAGTGTCAATATC | S |
| 9618R | GCCACACTCATAAGGGTTTGTT | S |
| 9685F | AAATCGCCCTACTATTGTC | S |
| 10088F | TGCCTAGAAGGAATAATA | S |
| 10467R | GGTGTTGTTAGTGGGTCGGA | S |
| 10747R | GGAGCCGGTTAGtGTgTAGAATAGGAA | S |
| 10966F | TCAATAGTMCTTGCYGCAGTACT | S |
| 11183F | AGCTTCCCTCATCCAAACCCCCTGAAGCTT | L,S |
| 11447R | GGTGTGTGAGCGCTCCTCGTTG | S |
| 11471F | AAcATACTAATtACaGCCCTCTA | S |
| 11718R | CCAATGGATAGCTGTTATCCTTTAAAAGTTGAG | L |
| 11759R | ACTTTTATTTGGAGTTGCACCA | S |
| 11826F | AAtATTGGTGCAACTCCA | S |
| 12108F | GACCCAAACATCAACCAATTCT | S |
| 12273R | TGCTTGGATGGCTGCTGTGTTGG | S |
| 12495F | GCAGCAGCAGGCAAATCAGCCCAA | S |
| 12576R | GCTGCTGTGTTGGCATCTGTT | S |
| 12820F | CAAGACATTCGAAAAATAGG | S |
| 12958F | ATTATCGAAGCCGCAAACAC | S |
| 13137R | AGTAGGGTGGGGTTATTTTCGT | S |
| 13415F | ATAAGCCAAAACCTAGCCCTTC | S |
| 13544R | AGGGGGAT RAGGAA RGAGAGG | S |
| 13592F | TCAATCACAATATATACACC | S |
| 13859F | GAACCCCATGTCTCAGGGTA | S |
| 13863F | GACCCCCTAAAACCTCAAGC | S |
| 14043R | TTTTCTTCTAAGCCTTCTCC | S |
| 14098F | CAACCAACCACACCACTAACAA | S |
| 14166R | GTTATGGCTGTAGTCCATGCAA | S |
| 14192R | TTGGGGTCATTAGTGCTTTTG | R,S |
| 14504F | ACCACTGGCATCATACTCCTAT | S |
| 14569F | CCATGAGGCCAAATATCATTCT | S |
| 14691F | ACTCTAATACGATTCTTCAC | S |
| 14824F | CTCAGACAAAATCGCCTTCC | S |
| 15091R | TGTGGKGGGGTGTTTAGTGGGT | S |
| 15299F | CTGAATTGGAAGCCAACCAGTAA | S |
| 15646F | GCATTACTGCTAGCCAACATGAA | S |
| 15982F | CCCTTGTTCACCATCCTCCGTGAAA | S |
| 16048F | GCTTCCCATGCATTTGGTAT | R,S |
| 16104F | ATGCTGACTCCCACCACATC | R,S |
| 16177F | ATCGCACCTACATTCC | S |
| 16228R | GTCCTACAAGCATGAATTAAATAAC | S |

Function Codes: L-LongPCR, R-Reamplification, S-Sequencing Primer
